# Combined treatment with artesunate and bromocriptine has synergistic anticancer effects in pituitary adenoma cell lines

**DOI:** 10.18632/oncotarget.17437

**Published:** 2017-04-26

**Authors:** Xin Wang, Qiu Du, Zhigang Mao, Xiang Fan, Bin Hu, Zhen Wang, Zhiyong Chen, Xiaobing Jiang, Zongming Wang, Ni Lei, Haijun Wang, Yonghong Zhu

**Affiliations:** ^1^ Department of Histology and Embryology, Medical School of Sun Yat-Sen University, Guangzhou, China; ^2^ Department of Neurosurgery and Pituitary Tumour Center, The First Affiliated Hospital of Sun Yat-Sen University, Guangzhou, China; ^3^ Department of Neurosurgery, The Fifth Affiliated Hospital of Sun Yat-Sen University, Zhuhai, China

**Keywords:** pituitary adenoma, synergistic treatments, miR-200c, Pten

## Abstract

Prolactinomas are the most prevalent functional pituitary adenomas. The preferred treatments for prolactinomas are dopamine agonists (DAs) such as bromocriptine (BRC), but DAs still have the challenges of tumor recurrence and drug resistance. This study demonstrates that the synergy of function and mechanism between artesunate (ART) and BRC inhibits prolactinoma cell growth *in vitro*. We found that low-dose ART combined with BRC synergistically inhibited the growth of GH3 and MMQ cell lines, caused cell death, attenuated cell migration and invasion, and suppressed the expression of extracellular prolactin. The induction of apoptosis after co-treatment was confirmed by immunofluorescent staining, assessment of caspase-3 protein expression, and flow cytometry. Expression of miR-200c, a carcinogenic factor in pituitary adenoma, was reduced following co-treatment with ART and BRC. This was accompanied by increased expression of the antitumor factor *Pten*. Transfection experiments with miR-200c analogs and inhibitors confirmed that miR-200c expression was inversely associated with *Pten* expression. We suggest that ART and BRC used in combination exert synergistic apoptotic and antitumor effects by suppressing miR-200c and stimulating *Pten* expression.

## INTRODUCTION

Hereditary genetic predisposition, endocrine factors, and specific somatic mutations may contribute to the development of pituitary adenomas. These common neuroendocrine neoplasms occur in almost 20% of the general population, and mainly cause compression and hormonal hypersecretion [1–5]. At autopsy, the incidence of pituitary adenoma reached 27% [6].

Currently, dopamine agonists (DAs) and somatostatin analogs are well-established treatments to suppress prolactin (PRL) and growth hormone (GH) hypersecretion [7]. Bromocriptine (BRC) is a DA that increases dopamine levels in the brain and thus reduces PRL secretion and tumor size. However, tumors have been shown to recur after the withdrawal of DAs, and DAs are associated with a dose-dependent increase in the risk of cardiac valve regurgitation, along with the induction of retroperitoneal and pulmonary fibrosis [7–10] and an impaired quality of life. Moreover, single chemotherapeutic agents can lose their potency over time as a result of increased drug resistance [11].

Artesunate (ART), a natural sesquiterpene lactone derivative of artemisinin, can be extracted from the Chinese herb *Artemisia annua*, and is well established as an effective antimalarial treatment [12]. Recently, ART was reported to exhibit antitumor effects [13, 14]. We previously demonstrated that ART inhibited pituitary adenoma cell proliferation, induced apoptosis, and reduced hormone synthesis and secretion [15, 16]. In the present study, we used GH3 and MMQ pituitary cell lines to investigate whether ART combined with BRC would have greater antitumor effects than single therapy with BRC, or even synergistically inhibit pituitary adenoma cell growth *in vitro*.

Our previous research also indicated that miR-200c was differentially expressed in pituitary adenoma specimens and MMQ cells, and that its expression likely contributed to pituitary adenoma pathogenesis [17]. Previous studies have demonstrated that the tumor suppressor gene phosphatase and tensin homolog (*PTEN*), was associated with miR-200 in tumor progression [18, 19]. Therefore, we also investigated whether miR-200c and its target gene *Pten* were involved in the synergistic anticancer effects of ART and BRC in pituitary adenoma GH3 and MMQ cell lines.

## RESULTS

### BRC and ART synergistically inhibited pituitary adenoma cell growth and induced cell death

**Figure 1:**
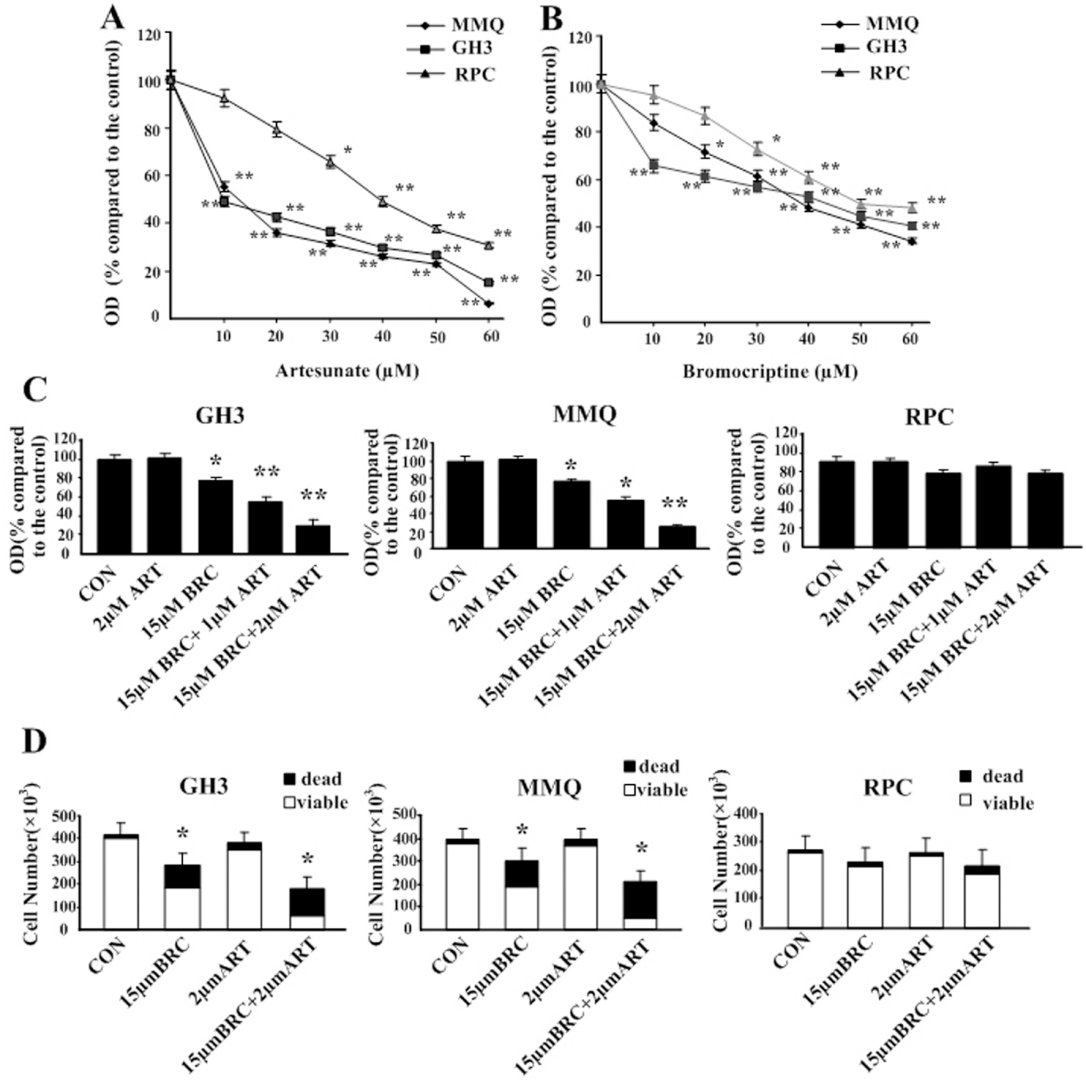
Artesunate (ART) and bromocriptine (BRC) synergized to inhibit pituitary adenoma proliferation and induce cell death **(A, B)** ART and BRC reduce pituitary adenoma cell proliferation. MMQ, GH3 and RPC cells were treated with **(A)** ART or **(B)** BRC for 48 h. Data shown are the mean ± SD of three independent experiments performed in triplicate. **(C)** After 48 h of treatment, cell viability was determined with a colorimetric MTT assay. **(D)** RPC, MMQ, and GH3 cells were treated with 15 μM BRC, with or without 2 μM ART, for 48 h. Trypan blue staining was used to detect cell death. Results are presented as **(C)** the percentage of untreated control cells (CON) ± standard error of six independent experiments or **(D)** absolute cell numbers. A single asterisk indicates *p* < 0.05; double asterisks, *p* < 0.01; and triple asterisks, *p* < 0.001 compared with controls and with single treatments. OD indicates optical density.

Treatment with ART (0–60 μM) or BRC (0–60 μM) alone significantly reduced the viable GH3 and MMQ cell numbers in a dose-dependent manner, as demonstrated by an MTT assay (Figure 1A and 1B). Cytotoxicity in rat pituitary cells (RPC) was also evaluated, and the results indicated that ART had a higher growth-inhibitory effect at various concentrations than BRC (Figure 1A and 1B). The IC_50_ values of ART in GH3 and MMQ cells were approximately 9.53 ± 4.12 μM and 18.37 ± 1.21 μM, respectively, and were approximately 21.89 ± 1.31 μM and 43.57 ± 3.31 μM for BRC.

Combined treatment with ART and BRC was then assessed at concentrations of the drugs that, alone, had little or no effect on viable cell numbers (Figure 1C). In GH3 and MMQ cell lines, 1 μM or 2 μM ART did not reduce the number of viable pituitary adenoma cells, whereas 15 μM BRC induced approximately 24% inhibition (*p* = 0.01). The combination of ART (2 μM) and BRC (15 μM) exhibited a synergistic effect and reduced the GH3 viable cell number by approximately 75% compared with controls (*p* = 0.002). This synergistic effect was even more pronounced in MMQ cells, in which the combination of 2 μM ART and 15 μM BRC reduced the viable cell number by more than 75% (*p* = 0.001). Of note, the combination of 1 μM ART and 15 μM BRC was less effective than the combination of 2 μM ART and 15 μM BRC (Figure 1C). Interestingly, the combined treatment had little or no effect on RPCs, in which the viable cell number remained greater than 80% (*p* = 0.87) (Figure 1C).

To determine whether the synergistic effects of ART and BRC observed in pituitary adenoma cell lines resulted from the induction of cell death, we manually scored the numbers of viable cells after Trypan blue staining. In MMQ cells, treatment with 15 μM BRC or 2 μM ART alone increased cell death by approximately 35%, whereas combination treatment induced cell death by over 75% (*p* = 0.01) (Figure 1D). A similar effect was observed in GH3 cells (*p* = 0.013). Thus, we demonstrated that co-treatment of GH3 and MMQ cells with 2 μM ART and 15 μM BRC synergistically inhibited proliferation and induced cell death.

### Combined ART and BRC treatment arrested pituitary adenoma cells in the G1 phase of the cell cycle

**Figure 2:**
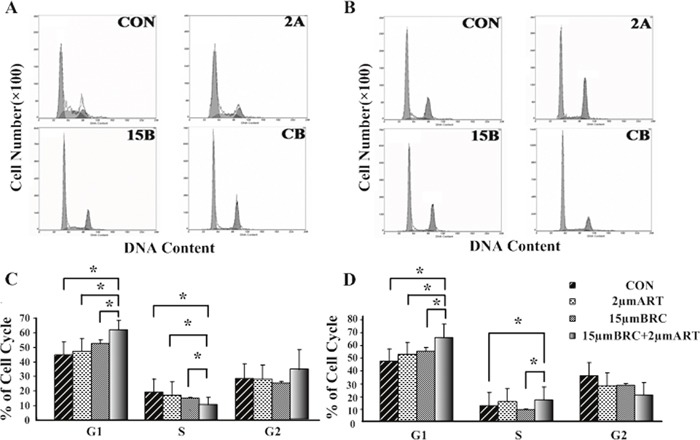
Combination treatment with ART and BRC arrested GH3 and MMQ cells in the G1 phase of the cell cycle GH3 **(A, C)** and MMQ **(B, D)** cells were treated with 15 μM BRC, with or without 2 μM ART. CON indicates negative control (untreated) cells, 2A indicates cells treated with 2 μM ART, and 15B indicates cells treated with 15 μM BRC. CB indicates combination treatment of cells with 15 μM BRC and 2 μM ART. After 24 h of treatment, the cell cycle distribution was analyzed by propidium iodide (PI) staining and flow cytometry. A single asterisk indicates *p* < 0.05.

To determine whether cell death induced by combined BRC and ART treatment was associated with antiproliferative effects caused by cell cycle disruption, fluorescence-activated cell sorting (FACS) was performed to assess the DNA content of GH3 and MMQ cells. ART did not arrest the cell cycle in GH3 and MMQ cells at the concentrations used, while BRC treatment slightly extended the G1 phase in GH3 cells (*p* = 0.033) but not in MMQ cells. ART and BRC in combination, however, resulted in G1 phase blockage in both cell lines (Figure 2). These cell cycle effects implied that combined treatment might inhibit cell proliferation and thus induce apoptosis.

### Combined treatment induced caspase-dependent apoptosis

**Figure 3:**
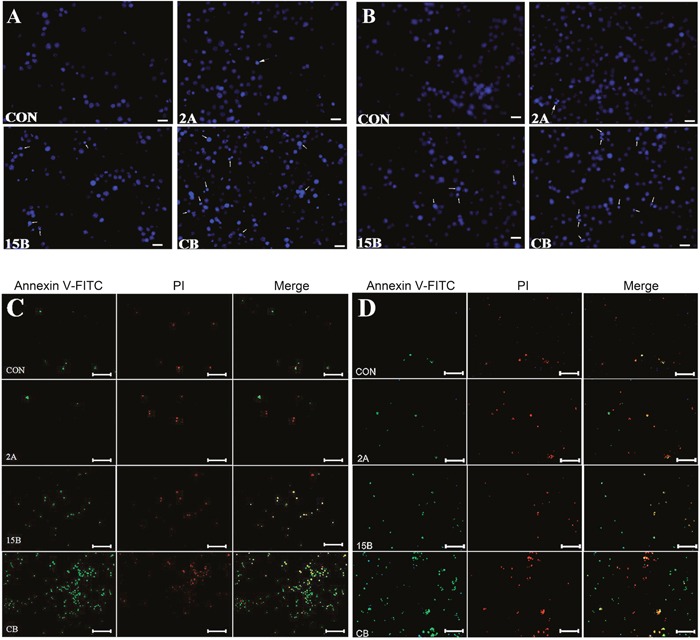
ART in combination with BRC induced apoptosis GH3 and MMQ cells were treated with 15 μM BRC, with or without 2 μM ART, for 24 h. **(A, B)** Nuclear fragmentation (white arrow) was detected by Hoechst 33342 staining. The numbers of apoptotic bodies are shown in GH3 **(A)** and MMQ **(B)** cells (white arrows). **(C, D)** Double staining with annexin-V and PI was performed. Annexin-V and PI were used to differentiate apoptotic cells from necrotic and normal cells; GH3 **(C)** and MMQ **(D)**.

**Figure 4:**
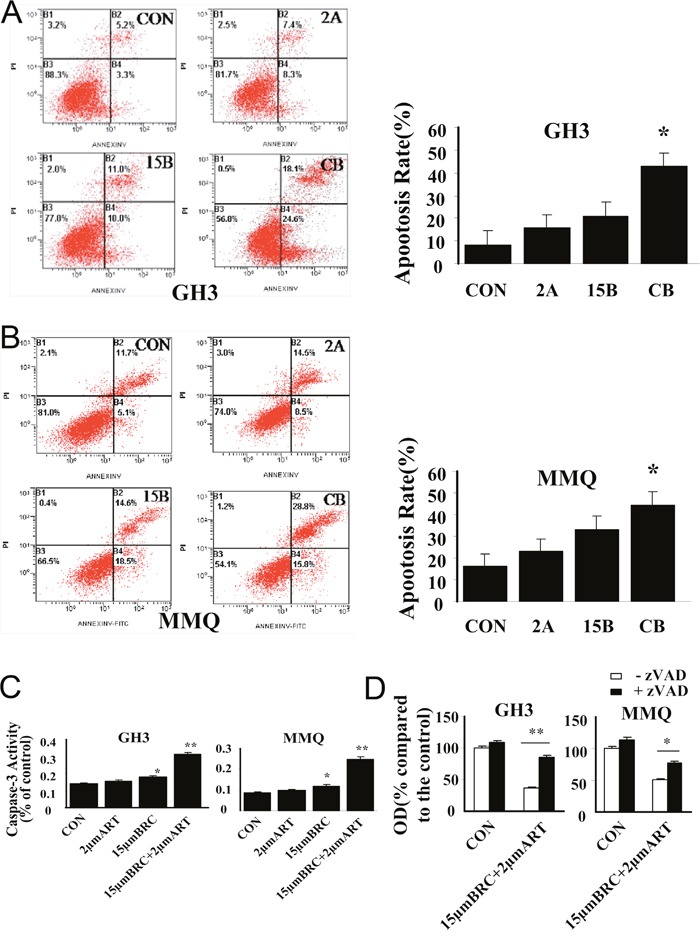
ART in combination with BRC activated caspase-3 and induced apoptosis Apoptosis in **(A)** GH3 and **(B)** MMQ cells was determined by annexin-V and PI staining and detected by flow cytometry analysis. **(C)** Caspase-3 activity assay detected caspase-3 activity. **(D)** The involvement of caspase activity in cell death induced by combination treatment with ART and BRC was analyzed by an MTT assay in the presence or absence of 10 μM caspase-3 inhibitor Z-Vad-FMK (zVAD). CON indicates negative control (untreated) cells, 2A indicates cells treated with 2 μM ART, and 15B indicates cells treated with 15 μM BRC. CB indicates combination treatment of cells with 15 μM BRC and 2 μM ART. Experiments were performed in triplicate, with one representative experiment illustrated. A single asterisk indicates *p* < 0.05, and paired asterisks indicate *p* < 0.01. Scale bar = 100 μm; -, negative; +, positive.

To characterize the mechanism of cell death induced by ART and BRC combination treatment, nuclear morphology and apoptosis markers were studied in GH3 and MMQ cells treated with these agents for 24 h (Figure 3). The combination of ART and BRC increased the number of cells displaying the nuclear morphology characteristic of apoptotic cell death, which includes pyknotic nuclei, nuclear chromatin condensation and nuclear fragmentation (Figure 3A and 3B). Furthermore, we performed annexin V and propidium iodide (PI) apoptosis assays to observe apoptosis by nuclear staining (Figure 3C and 3D) and FACS (Figure 4A and 4B). Co-treatment of GH3 and MMQ cells with ART and BRC dramatically increased the population of cells stained with annexin V and PI (*p* = 0.018 and *p* = 0.021); about 40% of cells were found to have entered apoptosis (Figure 4A and 4B). The apoptosis rates were higher for combination treatment than for treatment with ART and BRC alone. Combination treatment also increased caspase-3 in a synergistic manner compared with the single treatments (Figure 4C; *p* = 0.007 and *p* = 0.004). Furthermore, the pan-caspase inhibitor Z-Vad-FMK significantly inhibited the reduction in cell viability caused by the combined treatment (*p* = 0.008 and *p* = 0.013), confirming the classic caspase pathway as the predominant mechanism for GH3 and MMQ cell apoptosis (Figure 4D). These results indicated that the inhibitory effects on cell proliferation and viability induced by combination treatment might be attributable to apoptosis.

### Combination treatment attenuated migration and invasion and inhibited hormone secretion in pituitary adenoma GH3 and MMQ cell lines

**Figure 5:**
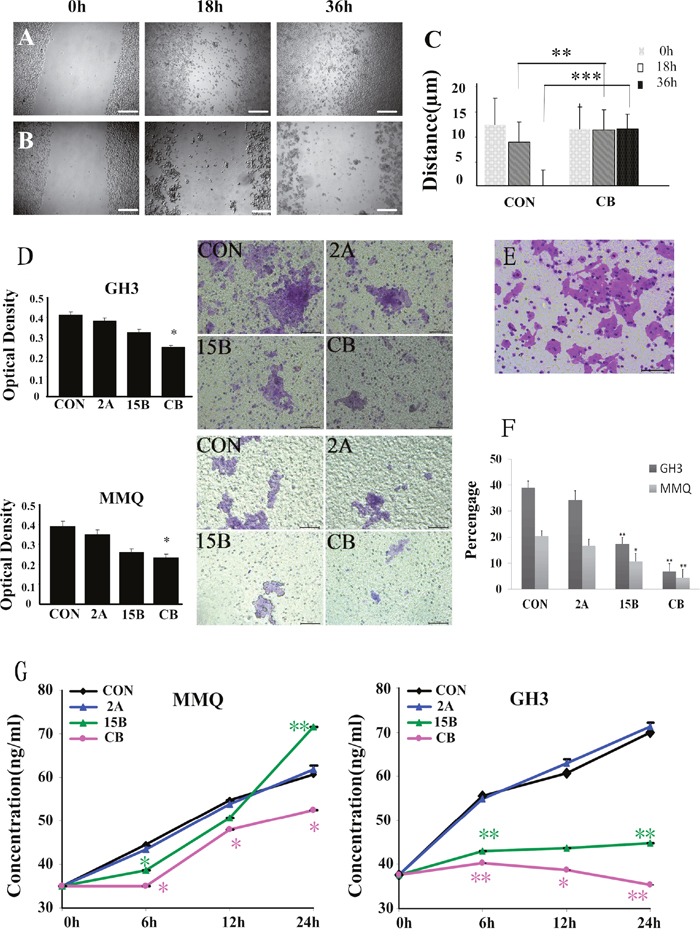
Wound-healing scratch assay in cells starved of serum for 24 h Bright field microscopy was used to determine the extent of closure under control conditions **(A)** compared with ART and BRC treatment **(B)** after 0, 18, or 36 h treatment of GH3 cells. Migration was quantified through measurement of the gap area before and after treatment **(C)**. Cell migration was calculated with Image Pro-plus software, and is represented as the difference in the scratch area before and after treatment. A representative result is shown. Scale bar = 200 μm. **(D)** Invasion assay of pituitary adenoma cells. GH3 and MMQ cells that migrated through the filter to the lower chamber were evaluated by crystal violet staining and the MTT assay, respectively. **(E)** Invasion assay of the human hepatoma HepG2 cell line as a positive control. Scale bar = 100 μm. **(F)** The percentage of cells that invaded through the Matrigel and non-migrant cells on the upper side of the filter. **(G)** ELISA was used to estimate extracellular PRL levels secreted by GH3 and MMQ cells. The experiment was repeated at least three times. A single asterisk indicates *p* < 0.05, and paired asterisks indicate *p* < 0.01.

To investigate whether combination treatment with ART and BRC inhibited cell migration and invasion, we performed wound-healing scratch assays (Figure 5A, 5B, and 5C) and invasion assays (Figure 5D, 5E and 5F). The combination treatment effectively reduced cell motility, indicating that co-treatment might inhibit the migration and invasion of pituitary adenoma cells. An ELISA assay also revealed a superior inhibitory effect of the combination treatment on the production and release of PRL in MMQ and GH3 cells (Figure 5G).

### Combination treatment induced cell apoptosis by downregulating miR-200c expression

**Figure 6:**
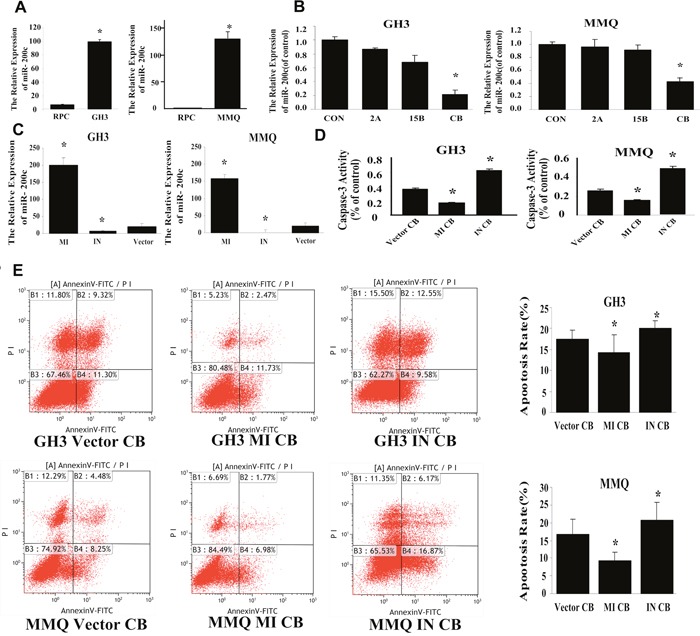
Combination treatment induced cell apoptosis by reducing miR-200c expression **(A)** Expression of miR-200c in RPC, GH3 and MMQ cells (*p* = 0.003). **(B)** qRT-PCR demonstrated that combination treatment specifically reduced miR-200c expression in GH3 (*p* = 0.013) and MMQ (*p* = 0.027) cells. **(C)** qRT-PCR was used to detect the transfection efficiency in GH3 and MMQ cells. MI indicates transfection with the miR-200c analog, IN indicates transfection with the miR-200c inhibitor, and Vector indicates the negative control. **(D)** Caspase-3 activity assay in transfected cells after combination treatment. **(E)** FACS was used to estimate apoptosis in transfected cells after combination treatment. Experiments were repeated at least three times; *p* < 0.05 versus control.

In previous studies, we found that miR-200c was markedly higher in the MMQ [17] and GH3 cell lines than in RPC cells (Figure 6A). We therefore investigated whether the combination treatment would up- or downregulate miR-200c expression in these cells. Real-time PCR analysis demonstrated that only combination treatment significant downregulated miR-200c expression in MMQ and GH3 cells (*p* = 0.003) (Figure 6B), and that combination treatment reduced the level of miR-200c in a synergistic manner.

To understand the effect of the combination treatment on the biological function of miR-200c, we transduced the GH3 and MMQ cell lines with a miR-200c analog or inhibitor. The transfection rates are shown in Figure 6C. In cells transfected with the analog, the levels of miR-200c were dramatically greater than the levels in vector control cells. Caspase-3 activity was assessed after combination treatment of transfected cells (Figure 6D), and the results indicated that overexpression of miR-200c reduced caspase-3 activity (*p* = 0.036, *p* = 0.021) while downregulation of miR-200c increased caspase-3 activity (*p* = 0.019, *p* = 0.011). When FACS was used to detect apoptosis in transfected cells after combination treatment, overexpression of miR-200c was shown to reduce apoptosis (*p* = 0.028, *p* = 0.035) (Figure 6E), while antagonization of miR-200c increased apoptosis after combination treatment (*p* = 0.014, *p* = 0.02). These analog and antagonist transfection experiments indicated that the combination treatment might induce apoptosis by downregulating miR-200c expression.

### Downregulation of miR-200c during combination treatment-induced apoptosis is accompanied by increased expression of *Pten*

**Figure 7:**
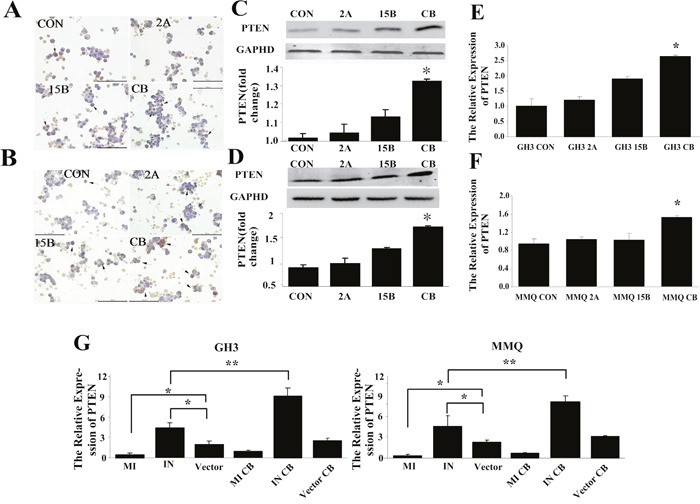
*Pten* was associated with miR-200c downregulation in combined treatment-induced apoptosis **(A-F)** Combination treatment with ART and BRC increased *Pten* expression in MMQ **(A, C, E)** and GH3 **(B, D, F)** cells. *Pten* mRNA and protein levels were determined by qRT-PCR **(E, F)**, immunocytochemistry **(A, B)**, and Western blot **(C, D)** analysis. Black arrows indicate positive cells; scale bar = 100 μm. **(G)** Expression of *Pten* mRNA in miR-200c-transfected cells detected by qRT-PCR. With combination treatment after miR-200c transfection, *Pten* mRNA decreased in analog-transfected cells and increased in inhibitor-transfected cells. All experiments were repeated three times, and representative results are shown. CON indicates control (untreated) cells. A single asterisk indicates *p* < 0.05 compared with untreated controls.

As in our previous study, we evaluated *Pten* expression by immunocytochemistry, Western blotting, and qRT-PCR (Figure 7). Immunocytochemistry revealed that PTEN was present in the cytoplasm (plurality) and nucleus (Figure 7A and 7B). Western blotting indicated that PTEN protein expression increased after combination treatment (Figure 7C and 7D), and qRT-PCR also demonstrated that the combination treatment stimulated *Pten* mRNA expression in GH3 (*p* = 0.012) and MMQ (*p* = 0.023) cells (Figure 7E and 7F).

To explore the regulatory relationship between miR-200c and *Pten*, we examined *Pten* expression after transfecting GH3 and MMQ cells with a miR-200c analog or inhibitor. As shown in Figure 7G, overexpression of miR-200c markedly reduced *Pten* mRNA levels (*p* = 0.014, *p* = 0.021), while miR-200c antagonization distinctly increased the expression of *Pten* mRNA (*p* = 0.022, *p* = 0.034). These results indicated that miR-200c negatively regulates *Pten* mRNA expression.

To investigate the regulation of *Pten* by miR-200c following combination treatment, we performed the co-treatment in GH3 and MMQ cells transfected with the miR-200c analog or inhibitor, and evaluated *Pten* expression relative to that in vector control cells. Following combination treatment, *Pten* expression decreased in analog-transfected cells (*p* = 0.027, *p* = 0.039) and increased in inhibitor-transfected cells (*p* = 0.030, *p* = 0.018) (Figure 7G). These observations suggested that the downregulation of miR-200c may be functionally important for the upregulation of *Pten* in combination-treatment induced apoptosis.

### *Pten* overexpression reduced miR-200c expression

**Figure 8:**
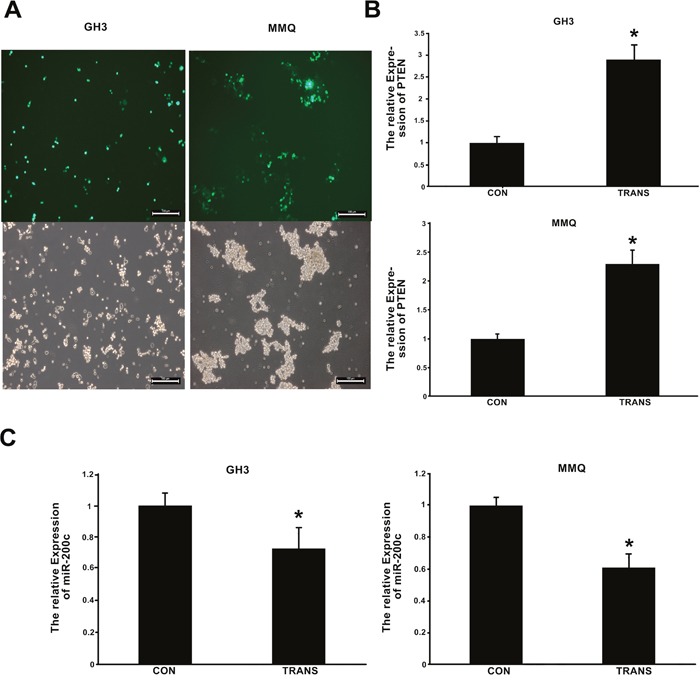
Expression of *Pten* and miR-200c 48 h after transfection of cells with pcDNA3.1/*Pten* and pcDNA3.1/*Egfp* plasmids **(A)** Green fluorescence was measured with a fluorescence microscope, and suggested that the transfection efficiency of the *Pten* plasmid was about 40-50%. **(B, C)** *Pten* and miR-200c expression 48 h after transfection with pcDNA3.1/*Pten* and pcDNA3.1/*Egfp* were determined by qRT-PCR. CON indicates cells transfected with pcDNA3.1/*Egfp*, and TRANS indicates cells transfected with pcDNA3.1/*Pten*. **(B)** *Pten* expression increased after transfection with pcDNA3.1/*Pten*. *p < 0.05. C, miR-200c expression decreased after transfection with pcDNA3.1/*Pten*. *p < 0.05.

The expression of *Pten* following transfection of cells with pcDNA3.1/*Pten* and pcDNA3.1/*Egfp* was determined by qRT-PCR, and was found to be greater following the former treatment (*p* < 0.01) (Figure 8B). The transfection efficiency of the *Pten* plasmid was shown to be about 40-50% (Figure 8A). In MMQ and GH3 cells, miR-200c expression was reduced after the cells were transfected with pcDNA3.1/*Pten* plasmid (Figure 8C).

## DISCUSSION

DAs have been used to treat prolactinomas for 35 years, and increasing recurrence rates have been reported in the past 10 to 15 years after withdrawal of treatment. Colao et al. evaluated the withdrawal of cabergoline in 200 patients (median duration of therapy: 36 to 48 months) and found that the recurrence rate of hyperprolactinemia after a median follow-up period of 12-18 months was 30% for microprolactinomas and 36% for macroprolactinomas [20]. Similarly, Kharlip et al. reported a recurrence of over 60% for hyperprolactinemia at 18 months [21]. A meta-analysis of 19 studies and a total of 743 patients indicated that only 21% of patients had persistent normoprolactinemia after DA withdrawal [22].

Besides recurrence, there are other challenges in the treatment of pituitary adenoma, including DA resistance and safety. The prevalence of DA resistance in prolactinomas, including macro- and microprolactinomas, was 25-50% for BRC and 5-15% for cabergoline [23]. Our previous report indicated that ART induced apoptosis and inhibited proliferation in GH3 and MMQ cells [15, 16]. In an effort to potentiate the inhibition of pituitary adenoma cell growth against a background of altered DA resistance and side effects, we evaluated ART in combination with low-dose BRC, and demonstrated that this combination treatment inhibited pituitary adenoma GH3 and MMQ cell growth. Furthermore, at the concentrations used in this work (2 μM ART with 15 μM BRC), the drugs had less influence on RPC cells.

BRC causes a time- and dose-dependent delay in the cell cycle, as shown by Johansen et al., who found that GH3 cells in S phase are most sensitive to BRC [24]. We found that combination treatment with ART and BRC arrested GH3 and MMQ cells in the G1 phase of the cell cycle, indicating that the antiproliferative effect of combination treatment may result from a cell cycle blockade.

Pituitary adenomas are distinguished by a series of phenotypic abnormalities, and although these fundamental defects manifest themselves as intractable disease, oncogenes and tumor suppressor genes typically involved in the pathogenesis of other tumors do not [25, 26]. *PTEN* is frequently mutated or deleted in numerous tumors, especially in endometrial carcinoma [27]. Whether its activity is lost by mutation, deletion, or promoter methylation silencing at high frequency, as is the case in many primary and metastatic human cancers, *PTEN* downregulation usually has the same result: persistent AKT signaling that contributes to tumor formation [28, 29]. The majority of studies have demonstrated that *PTEN* is downregulated in pituitary adenomas rather than mutated. Qi et al. [30] found that *Pten* expression in the pituitary glands of rats decreased after the rats were exposed to electromagnetic pulses. Tena-Suck et al. [31] analyzed the immunoexpression of PTEN in 45 cases of pituitary adenoma, and noted that PTEN expression was higher in nonfunctional hypophysis adenomas than in functional adenomas. Chen et al. [32] demonstrated that autocrine PRL production is induced by the PTEN–PI3K–AKT pathway, while Musat et al. [33] reported that PTEN expression was reduced and the AKT pathway was activated with cell cycle changes in pituitary tumors. Our results demonstrated that the protein and mRNA levels of *Pten* increased after combination treatment of MMQ and GH3 cells.

MiR-200c is involved in the tumorigenesis and progression of many cancers [34, 35]. In our previous study, we found that the expression of miR-200c was higher in human pituitary adenoma samples than in normal pituitary samples. To investigate whether miR-200c was involved in combination treatment-induced apoptosis, we evaluated the expression of miR-200c in GH3 and MMQ cells. MiR-200c was reduced in a synergistic manner after combination treatment, and overexpression of miR-200c suppressed combination treatment-induced apoptosis, while antagonization of miR-200c increased the rate of apoptosis after combination treatment. These results implied that miR-200c is involved in the resistance to apoptosis induced by combination treatment. In a further exploration of this mechanism, we determined that miR-200c expression was inversely associated with *Pten* expression in GH3 and MMQ cells after combination treatment. This outcome indicated that ART and BRC function synergistically in pituitary adenoma cells through enhanced inhibition of miR-200c by binding *Pten*.

In summary, our study demonstrated that the synergistic inhibitory effects of ART and BRC on GH3 and MMQ cells resulted from the induction of apoptosis. Combination treatment also attenuated migration and invasion, and reduced hormone secretion in these cells. The reduction in miR-200c expression is attributed to apoptosis induced by combination treatment, which also increased the expression of *Pten* (which was negatively associated with miR-200c levels and positively associated with combination treatment-induced apoptosis). The combination of ART with BRC has important future clinical potential, which may be applied as an adjuvant therapy to improve the therapeutic efficacy of prolactinoma treatment.

## MATERIALS AND METHODS

### Chemicals and reagents

BRC (as 2-bromo-α-ergocryptine methanesulfonate salt) was purchased from Novartis. ART for injection was obtained from Guilin Pharma (China). Rat PRL (rPRL), anti-rPRL antibody, and anti-rat GH antibodies were purchased from CST (MA, USA).

### Cell culture

The rat pituitary adenoma cell lines GH3 and MMQ were purchased from the Xie-he Cell Bank, China. Cells were cultured in complete F12 medium (Sigma, US) supplemented with 15% horse serum, 2.5% fetal bovine serum (FBS), 5 U/mL penicillin, and 5 μg/mL streptomycin (Invitrogen, CA, USA).

Rat pituitary cells (RPC) were purchased from Ya ji Company, China, and were cultured in RPMI1640 medium (Invitrogen) supplemented with 10% FBS. All cultured cells were maintained at 37°C in a humidified atmosphere of 5% CO_2_.

### Cell growth and viability

Cells in log phase were washed three times with serum-free culture medium and seeded (GH3 cells, 20,000–30,000 cells/well; MMQ cells, 30,000–40,000 cells/well; and RPC cells, 30,000–40,000 cells/well) into 96-well plates. The next day, cells were treated with different agonists. Cells were incubated in MTT solution for approximately 4 hours. For GH3 and MMQ cells, 100 μl acidified isopropyl alcohol was added to each well, and the optical density was determined at 570 nm using a spectrophotometer (Tecan, Austria). The formazan crystals from RPC cells were dissolved in 100 μL of DMSO, and the optical density was determined at 490 nm. Cell viability was assessed by Trypan blue exclusion; viable and nonviable cells were manually counted. The results are reported as the mean ± standard deviation (SD) of at least three independent experiments.

### Caspase activity assay

About 20,000 cells were treated for 48 h with the indicated concentration of ART or BRC, or a combination of both. Caspase-3 activity was assessed in 10 μL of total cell lysate with a Caspase-3 Activity Assay kit (BestBio, China), with spectrophotometric measurement at 405 nm. Per the manufacturer's recommendations, the total protein concentration was measured with Coomassie blue G250 staining (BCA; BestBio, China). Cells treated with culture medium were used as a positive control. All experiments were performed in triplicate.

### Cell cycle analysis

Cells were seeded in six-well plates at a density of 1 × 10^6^ cells/well, harvested after 48 h of treatment, and fixed with 500 μL of 70% ethanol overnight at 4°C. Prior to cell cycle analysis, cells were washed twice with phosphate-buffered saline (PBS), resuspended in 100 μL of DNase-free RNase A, and incubated for 30 min at 37°C. Next, 100 μL of PI solution was added and cells were incubated for 30 min at 4°C in the dark. The distribution of the cellular DNA content was analyzed by FACS (EPICS XL-MCL, Beckman Coulter, Roissy, France). All experiments were performed in triplicate.

### Apoptosis analysis

Cells were seeded in six-well plates at a density of 1 × 10^6^ cells/well and incubated for 24 h before treatment. Hoechst 33342 and Annexin V-FITC/PI staining were used to detect apoptosis. All experiments were performed in triplicate.

### Scratch assay

Cells were seeded in 24-well poly-D-lysine-treated plates at a density of 1 × 10^5^ cells/well until confluence of 80–90% in a monolayer was reached. The monolayer was carefully scratched with a sterile 10-μL pipette tip across the center of the bottom of the well, and a second straight line was scratched perpendicular to the first to create a cross in each well. After the scratching, the cells were gently washed twice with PBS so that detached cells could be removed, and the remaining cells were cultivated for 48 h in culture medium with treatments. The scratched area was observed again, and stained monolayers were photographed in triplicate under microscopy. All experiments were also performed in triplicate.

### Vector construction

The 3′-UTR of the *Pten* mRNA (containing the miR-200c binding site) was amplified by PCR and cloned into the *BamHI/EcoRI* site of a pcDNA3.1(+) vector (Zoonbio) to construct the luc-*Pten* plasmid.

### Enzyme-linked immunosorbent assay (ELISA)

Cell culture supernatants were centrifuged for 20 minutes at 1000 g, and samples were stored at −80°C. PRL levels in the supernatants were determined with a rat-specific ELISA kit (USCNK) for PRL, according to the manufacturer's instructions. The concentrations of PRL in the samples were determined through comparison of the optical densities of the samples with the standard curve, and were expressed as ng/mL and percent of control.

### Invasion assays

Cell migration assays were performed in 24-well Transwell plates with 8-μm pores (Corning, Santa Clara, CA, USA). Stable GH3 and MMQ cells (5 × 10^5^ cells per well) were seeded in the upper culture chambers with serum-free F12 medium, while growth medium with 10% FBS was added to the lower chambers. After 48 h of incubation, both cells that invaded through the Matrigel and non-migrant cells on the upper side of the filter were counted and assessed. Filters were fixed with 4% formaldehyde for 15 minutes, then stained with 0.1% crystal violet for 15 min. Three random fields were counted, and the quantified results are presented as the mean ± SD.

### Western blotting

Cold PBS was used to rinse cells twice after treatment. Cells were lysed with cell lysis buffer, and the protein content in the supernatant was determined with a BCA protein assay. For Western blot analysis, equal amounts of total protein were mixed with 6× SDS sample buffer and incubated at 100°C for 10 min, and aliquots of 30 μg protein in total were subjected to electrophoresis on 8–12% SDS-PAGE gels. Proteins were transferred to polyvinylidene difluoride membranes (Millipore Co., MA, USA), which were rinsed twice with Tris-Buffered Saline and Tween 20 (TBST), and incubated with 5% non-fat milk (in TBST) for 1 hour at room temperature. Following overnight incubation of the membranes with the primary antibodies (anti-GAPDH, 1:1000; anti-PTEN 1:1000) at 4°C, three washes with TBST were performed and the membranes were incubated with an HRP-conjugated secondary antibody at room temperature for 2 h. Following three washes with TBST, the antibody-bound proteins were detected with a Super ECL Plus Detection Reagent (Applygen Technologies Inc., China). The protein band density was determined with ImageJ 1.38X software.

### Immunocytochemistry

Cells were seeded into six-well poly-D-lysine-treated plates at 5 × 10^4^ cells/well and allowed to adhere before being fixed with 4% paraformaldehyde for 15 minutes, washed twice with ice-cold PBS, blocked with 10% goat serum for 30 min, and incubated overnight with a diluted PTEN antibody (1:100, Bioss, China) in PBST at 4°C. Next, cells were washed three times in PBS for 5 min per wash and incubated with the secondary antibody in PBST for 1 h at room temperature in the dark. After the plates were rinsed with PBS, PI-conjugated goat anti-rabbit IgG (1:100, Bioss, China) was added and the cells were incubated with 0.1–1 μg/mL Hoechst 33342 for 1 min. The Streptavidin-Biotin Complex method was performed according to the manufacturer's instructions. Fixed cells were visualized and images were captured with an Axio Observer Z1 (Carl Zeiss, Inc).

### qRT-PCR

Primers for the analysis of RNA expression were designed by Primer-BLAST (www.ncbi.nlm.nih.gov). Mixtures of 1 μg total RNA, 50 nM reverse primer, 2 units of RNase inhibitor (Toyobo, Osaka, Japan), 5 units of M-MLV reverse transcriptase (Toyobo) and 0.5 μM dNTP were used for each RT reaction. qRT-PCR was performed in 20-μL reactions with the Bio-Rad S1000 detection system (Toyobo).

### Transfection of cells by electroporation

Cells were collected by centrifugation at 500 g for 5 min at 4°C and resuspended in the electroporation medium at a concentration of 5 × 10^6^ cells/mL. Next, 25μg of pcDNA3.1/*Pten* and pcDNA3.1/*Egfp* plasmid DNA were added separately in a volume of up to 80 μL per cuvette (Sigma) of cells [36]. Cells were incubated on ice for 10 min before electroporation, and cell-line-specific parameters were set on the electroporation device (280 V, and a single electric pulse of 10 ms). Cuvettes were removed and placed immediately on ice for 5 min. Electroporated cells were transferred to a 100-mm culture dish. The cuvettes were washed three times with fresh growth medium, and the washing solution was also added to the culture dish.

### Statistical analysis

Data are expressed as the mean ± SD from at least three independent experiments. In all experiments, the significance of the difference between two groups was calculated with Tukey's test after one-way analysis of variance in SPSS 13.0 software. *P* < 0.05 was considered statistically significant.
